# *In Situ* Formed Phase Transited Drug Delivery System of Ketoprofen for Achieving Osmotic, Controlled and Level A *In Vitro In Vivo* Correlation

**DOI:** 10.4103/0250-474X.49096

**Published:** 2008

**Authors:** A. K. Philip, Kamla Pathak

**Affiliations:** Department of Pharmaceutics, Rajiv Academy for Pharmacy, Mathura, Uttar Pradesh-281001, India

**Keywords:** *In situ*, phase-transited, osmotic, correction, *in vivo*, glass holders

## Abstract

A dry process induced phase transited, non disintegrating, controlled release, *in situ* formed asymmetric membrane capsular system for poorly water soluble drug, ketoprofen, was developed and evaluated both *in vitro* and *in vivo* for osmotic and controlled release of the drug. *In situ* formed asymmetric membrane capsules were prepared using fabricated glass capsule holders via dry, phase inversion process. Effect of varying osmotic pressure of the dissolution medium on drug release was studied. Membrane characterization by scanning electron microscopy showed an outer dense region with less pores and an inner porous region for the prepared asymmetric membrane. *In vitro* release studies and statistical test for all the prepared and marketed formulation were done at *P* >0.05. The drug release was found to be independent of the pH, but dependent on the osmotic pressure of the dissolution medium. *In vivo* pharmacokinetic studies showed a level A correlation (R^2^>0.99) with 39.24 % relative bioavailability compared to immediate release tablet of ketoprofen. Excellent correlation achieved suggested that the *in vivo* performance of the phase transited *in situ* formed AMCs could be accurately predicted from their *in vitro* release profiles and could a means for controlled delivery of drugs with varying solubility.

There has been increasing interest in the development of osmotic devices in the past two decades, and various osmotic pumps have been reviewed^1^. The elementary osmotic pump (EOP) was first introduced by Theeuwes in the 1970s^2^. However, this type of EOP was only suitable for the delivery of water soluble drugs. To overcome the limit of EOP, a push-pull osmotic tablet was developed in the 1980s. The push-pull osmotic tablet had two disadvantages: (1) the tablet core was prepared by compressing 2 kinds of compartments together, a complex technology as compared with that of EOP, and (2) after coating, a complicated laser-drilling technology was used to drill the orifice next to the drug compartment^3^. To avoid sophisticated techniques of all osmotic tablet systems, monolithic osmotic tablet system was proposed and studied^4^. Osmotic tablets with an asymmetric membrane coating, which can achieve high water fluxes, have been described^5^. The asymmetric membrane capsule (AMC) prepared either by wet^6^ or dry process^7^ are also examples of a single core osmotic delivery system, consisting of a drug containing core surrounded by an asymmetric membrane. One of the advantages of an asymmetric membrane is the higher rate of water influx, allowing the release of drugs with a lower osmotic pressure or lower solubility. Wet process AMCs both as delayed release system^8^ and conventional controlled release have been shown to be a good delivery system in achieving level A *in vitro in vivo* correlation (IVIVC)^9^. However, no such study has been reported for *in-situ* formed AMCs.

Ketoprofen [(RS)-2-(3-benzoylphenyl)propionic acid is an important non steroidal antiinflammatory drug (NSAID), effectively used in treatment of rheumatoid arthritis, osteoarthritis^10^ and musculoskeletal disorders^11^. Because of its short elimination half-life (4.2 h) which requires multiple dosing to achieve and maintain therapeutic concentration and poor aqueous solubility, hazards of adverse gastrointestinal (GI) reactions like gastric irritation, peptic ulceration and bleeding can occur. Development of oral sustained release formulations with a high level of IVIVC of this drug is highly desirable in order to achieve improved therapeutic efficacy and patient compliance.

Therefore, the aims of this work were to develop *in situ* formed phase-transited drug delivery system to deliver ketoprofen in a controlled manner, and to evaluate the *in vivo* performance of the prepared *in situ* formed phase transited drug delivery system.

## MATERIALS AND METHODS

Ketoprofen was obtained from Sun Pharmaceuticals Pvt Ltd, Gujarat, India. 3-benzoylbenzoicacid used as an internal standard for high performance liquid chromatography (HPLC) was synthesized in the laboratory from benzoic acid AR grade (Qualigens Fine Chemicals, Mumbai, India). Sodium di-hydrogen phosphate and di-sodium hydrogen phosphate (both analytical reagent grade) were purchased from S. D. Fine Chemicals, Mumbai, India. Ethylcellulose (EC, 50 cps), acetone, glycerin, and ethyl alcohol were procured from Qualigens Pvt Ltd, Mumbai, India. Sodium chloride (NaCl) from Merck India, New Delhi and hard gelatin capsules (#01) from Warner Lambert, New Delhi, India were purchased from C. N. Chemicals, Uttar Pradesh, India. Acetonitrile and methanol (HPLC grade) were procured from (Ranbaxy Fine Chemicals, Mumbai, India). Solvents of reagent grade and double-distilled water were used in all experiments. Wistar rats were purchased from the Central Drug Research Institute (Lucknow, Uttar Pradesh, India) and were housed in the animal house at the Department of Pharmacology, Rajiv Academy for Pharmacy, Mathura, Uttar Pradesh, India. The *in vivo* pharmacokinetic studies were conducted after prior approval from the institutional animal ethical committee (IAEC/RAP/1561).

### Solubility studies:

The kinetics of osmotic drug release is directly related to the solubility of drug within the formulation. Assuming the capsule formulation to consist only of the pure drug, the fraction of drug released with zero order kinetics is given by Eqn. 1^12^,^13^, F(z)= 1-S/ρ, where, F(z) is the fraction released by zero-order kinetics, S is the drug's solubility (g/cm^3^), and ρ is the density (g/cm^3^) of the drug. In general, drugs with a solubility of 0.05 g/cm^3^ would be released with 95% zero order kinetics according to Eqn. 1. However, zero order release rate would be slow due to the small osmotic pressure gradient. Conversely, highly water soluble drugs would demonstrate a high release rate that would be zero order for only a small percentage of the initial drug load. Therefore, to assess the solubility of the drug in various dissolution mediums, saturated solutions of the drug were prepared in 0.1N HCl, phosphate buffer pH 7.4 and double distilled water without and with 10, 15, 20, 25 and 30 mg citric acid in a closed container at 37°. Excess amounts of the drug were added to ensure saturation and the solutions were equilibrated for 24 h. The saturated solutions were filtered and concentration determined by UV spectrophotometer (Shimadzu 1700, Tokyo, Japan) at 260.5 nm after suitable dilutions. Density of the drug was determined by pycnometer (Jindal Scientific Industries Pvt. Ltd., Ambala, India).

### Preparation of *in situ* formed phase transited AMCs inside hard gelatin capsules:

Empty colorless conventional hard gelatin capsules (#01) were taken and the body and cap separated and put on individual fabricated glass capsule holders. Holders with capsule body and cap were rotated at 50 rpm and a coating solution of varying proportions (10%, 15%, and 20% w/v) of EC and glycerol (8% w/v) in acetone and ethanol were poured into the cap and body. With holders still rotating the coating solution was evaporated partially using a hair drier after which, capsules were kept at ambient room temperature for 24 h (holders still rotating) to facilitate further drying. This process resulted in phase inversion which was controlled by the rate of evaporation of a more volatile solvent. Asymmetric membranes formed inside conventional hard gelatin capsules were then filled manually with a constant drug loading (200 mg, after passing it through a 100 mesh sieve and regulating the particle size to 120 μm), previously mixed with an osmotic agent (NaCl) in a polythene bag. NaCl was used as an osmogen as ketoprofen was found to be osmotically inactive^14^. Filled hard gelatin capsules were sealed with a solution of EC in ethanol. Ingredients in the capsular system are listed in Table 1. Since drug solubility was expected to be a decisive factor for success of *in situ* formed phase transited AMCs, drug release from this drug delivery system was further studied by examining the influence of citric acid, considered to be a solubility enhancer for the drug (F11). For administration to rabbits, the optimized formulation was filled with a drug loading of 0.278 mg of ketoprofen/kg body weight of the rabbit (US FDA recommends using a factor of 12 for converting human dose to equivalent rabbit dose), mixed with 0.315±0.36 mg NaCl and 0.174±0.30 mg citric acid in a polythene bag, were manually filled inside the capsule.

**TABLE 1:** COMPOSITION OF THE NINE FORMULATIONS (F1 TO F9) ALONG WITH F11

Variables	Formulation code									

	F1	F2	F3	F4	F5	F6	F7	F8	F9	F11*
EC** (% w/v)	10	10	10	15	15	15	20	20	20	15
NaCl*** (mg)	0	50	100	0	50	100	0	50	100	50
Glycerol(% w/v)	8	8	8	8	8	8	8	8	8	8
Acetone (% v/v)	50	50	50	50	50	50	50	50	50	50
Ethanol (% v/v)	30	30	30	30	30	30	30	30	30	30
Citric Acid (mg)	0	0	0	0	0	0	0	0	0	30

* F11- formulation with citric acid

** EC- ethylcellulose

*** NaCl-sodium chloride

### Scanning electron microscopy (SEM):

Asymmetric membranes obtained before and after complete dissolution of core contents were examined for their porous structure using Jeol 6100 SEM (Jeol, Tokyo, Japan). After dissolution, asymmetric membrane structures were dried at 50° for 8 h and stored in dessicator before examination. Asymmetric membranes were sputter coated for 5 to 10 min with gold by using fine coat ion sputter and examined under SEM.

### *In vitro* drug release:

*In vitro* cumulative drug release from the prepared formulations (n = 6) was studied by using British Pharmacopoeia (BP) paddle type apparatus (rotating speed 75 rpm at 37±0.5°). The dissolution medium was 0.1N HCl as simulated gastric fluid (SGF, 900 ml, pH 1.2) for the first 2 h, followed by phosphate buffer as simulated intestinal fluid (SIF, 900 ml, pH 7.4) for the rest of the experiment. One milliliter of the sample was withdrawn at specified time intervals and suitably diluted by fresh dissolution medium and analyzed at 260.5 nm.

### Statistical analysis:

Release profiles up to t_50%_ of ketoprofen from all formulations (n=6) in dissolution medium were statistically compared with the marketed formulation of ketoprofen by Dunnett's Multiple Comparison Test (Instat software, Graphpad Software Inc, San Diego, CA). Best formulation amongst the formulations was chosen after pair wise comparison using dissimilarity factor (f_1_) and the formulation with the lowest f_1_ value but with Fickian diffusion was selected as the best formulation.

### Effect of varying osmotic pressure:

In order to confirm the mechanism of ketoprofen release, release studies of the optimized formulation were conducted in a media providing greatest sink condition, but of different osmotic pressure. To increase the osmotic pressure of the dissolution medium (SIF), NaCl (osmotically effective solute) was added, and the pH was adjusted to 7.4±0.5. Release studies were performed in 900 ml of phosphate buffer pH 7.4 using BP dissolution apparatus II (75 rpm). Two methods were employed, the first was the direct measurement of the ketoprofen in the dissolution medium at predetermined time intervals, and the second was residual analysis method (to reduce the effect of any chance interference of the ketoprofen by NaCl). In residual analysis method, the formulation undergoing dissolution was withdrawn from the vessel at predetermined intervals and cut open to dissolve the contents into 250 ml SIF. One milliliter of the sample was taken and suitably diluted and analyzed at 260.5 nm to determine the residual amount of drug in each AMC. Results were found to be comparable with both the methods.

### *In vivo* study conditions:

The experimental conditions were similar to the work described^15^. Twenty two rabbits were included in the study, which were divided into 3 groups. Group 1 consisted of 10 rabbits (5 males and 5 females) for pooled blood sample collection. Group 2 had 6 rabbits (3 male and 3 female) for *in vivo* pharmacokinetic studies of the test formulation and group 3 had 6 rabbits (3 male and 3 female) for *in vivo* pharmacokinetic studies of the reference formulation. The body weights of rabbits were determined before the start of the experiment.

### High performance liquid chromatography (HPLC) conditions:

Assay validation was done using Cecil 4200^®^ HPLC system. Instrumentation of HPLC system consisted of Cecil CE4100^®^ HPLC dual piston short stroke pump and a Cecil 4200^®^ UV-Visible detector was set at 260.5 nm for ketoprofen (Cecil instrument Ltd, England). The mobile phase consisted of acetonitrile:water:phosphate buffer (pH 3.5, 43:55:2 v/v/v), filtered and degassed under reduced pressure and pumped at 1 ml/min through C18 (Thermo Electron Corporation^®^, England) 250×4.6 mm column with 5 μ packing, with a typical pressure of 67±0.05 bars.

### Validation parameters:

The method of specificity was assessed by comparing the chromatograms obtained from the drug to its respective internal standard and with those obtained from the blank. Linearity, range, limit of quantification and limit of detection were obtained from the standard concentrations (0.3, 2, 4, 6, 8, 10 μg/ml) which in turn were obtained from the stock solution(s). Each concentration was prepared six times (n=6). The limit of quantitation (LOQ) was the lowest concentration assayed where the signal/noise ratio was at the least 10:1 and the limit of detection (LOD) was defined as a signal/noise ratio of 3:1. The accuracy, precision and recovery in plasma assay validation involved quality control (QC) concentrations prepared from newly prepared spiked stock solution of ketoprofen (1 μg/ml, 5 μg/ml and 10 μg/ml). The QC samples were divided into 0.1 ml aliquots in centrifuge tubes and stored at -70° before use. Intra day and interday variability were tested with 12 replicates of each QC control concentration. Means, standard deviations and coefficient of variation were calculated by standard methods. Recovery test were performed by adding known amounts of respective stock solution of ketoprofen to the sample with known content and preparing solutions with the respective mobile phase. The percentage of recovery was calculated by comparing the determined amount of these standards with the added amount.

### Route of administration and withdrawal of blood samples:

The rabbits were taken as per the phases divided for *in vivo* pharmacokinetic studies. The rabbits were administered the reference and the test formulations by the help of a gastric feeding tube after placing them in a restraining device (rabbit holder). This feeding tube was of 7 mm diameter which, had been bent to approximate the pharyngeal curvature. A mouth speculum was used to administer the capsules. The mouth speculum was a stainless steel rod which acted as a tongue depressor and had a centrally placed hole. Care was taken that the tube did not enter the trachea or puncture the esophagus or stomach which was evident as no violent reaction (coughing, gasping- which usually follows on accidental introduction of the tube into the larynx or trachea) were seen. For withdrawal of blood samples, the rabbits were anesthetized by subcutaneous injection of 25% urethane-physiological saline (4 ml/kg). A fine cannula hypodermic needle (0.8 mm) with syringe attached to it was inserted into a right femoral artery to facilitate the sampling of blood for drug analysis. The syringe was detached from the needle and the cannula closed with the cap to prevent clotting of the blood. To further ensure that clotting of blood did not take place the cannula before closing was flushed with 10% v/v of heparin/normal saline solution. The needle was kept inside the artery by means of leucoplast tape. At perquisite time periods, 2 ml of blood sample(s) were withdrawn through the cannula into heparinized glass vials and centrifuged at 3000 rpm for 10 min to obtain 1±0.14 ml of the plasma and frozen (-20°) until analyzed. Blood samples were taken at 0, 1, 2, 3, 4, 5, 6, 7, 8, 9, 10, 24 and 30 h for the test formulation and reference formulation of ketoprofen. To maintain homeostasis of the rabbits, an injection of same volume of physiological saline was given via the ear vein.

### Ketoprofen determination in rabbit plasma:

The determination of ketoprofen rabbit plasma was carried out by taking 1±0.14 ml aliquots of plasma, which were pipetted into a 15 ml centrifuge tube. Plasma drug mixture was prepared (100 μg) by adding 100 μg of acetonitrile, 200 μg of internal standard, 500 μg of a 2.5 M O-phosphoric acid solution in a 10 ml glass tube and vigorously shaken on a vortex mixer for 20 sec. The samples were then centrifuged at 3000 rev/min for 10 min. The organic layer was transferred to a 10 ml centrifuged tube and evaporated to dryness under stream of dry nitrogen at 37°. The residue was reconstituted in 250 μg of respective mobile phase. An appropriate aliquot (20-75 μg) was then injected directly into the loop injector.

### Pharmacokinetics and statistical analysis:

The plasma concentration time data of ketoprofen was fitted in Quick-Cal software (Plexus Supporting Services, Ahmedabad, India) and the pharmacokinetic parameters calculated. Mass balance model dependent technique (Wagner Nelson) was also used to calculate the absorption parameter. The area under the concentration-time curve (AUC_0-t_) was determined using the trapezoidal method. AUC_t-∞_ was calculated by dividing the last recordable plasma concentration over elimination rate constant. C_max_ and T_max_ were determined through the observation of individual animal drug concentration versus time curves. Relative bioavailability was determined by using the Eqn. 2, F_r_ =AUC_test_/AUC_std_×Dose_std_/Dose_test_×100..(2), to compare the main parameters of the different dosages forms, a two sided unpaired t-test was conducted with microsoft excel 2003. Statistical significance was tested at *P*<0.01.

### *In vitro in vivo* correlation (IVIVC):

The USP Biopharmaceutics subcommittee has established the four categories to define the correlation between *in vitro* dissolution and *in vivo* absorption namely Level A, B, C and D. Level A correlation is the highest category of correlation and is most applicable to modified release systems. With this correlative procedure, the specially designed AMCs *in vitro* dissolution curve was compared to the *in vivo* absorption rate after suitable deconvolution. The correlation was demonstrated after plotting the fraction absorbed *in vivo* versus the fraction release *in vitro*.

## RESULTS AND DISCUSSION

Solubility studies showed that ketoprofen had varying solubility in the different mediums studied, 0.1 N HCl (6.1×10^−6^ g/cm^3^), phosphate buffer pH 7.4 (12.62×10^−3^ g/cm^3^), double distilled water (8.21×10^−3^ g/cm^3^) and with 10 mg citric acid (25.33×10^−3^ g/cm^3^), 15 mg citric acid (30.12×10^−3^ g/cm^3^), 20 mg citric acid (46.12×10^−3^ g/cm^3^), 25 mg citric acid (49.87×10^−3^ g/cm^3^), and 30 mg (56.65×10^−3^ g/cm^3^). The density of ketoprofen was found to be 0.2717 g/cm^3^. The experimental values of the F(z) suggested that, in order to increase the rate at which zero order release kinetics is achieved by the fraction of drug undergoing dissolution, an external agent (buffering agent) needs to be incorporated in the formulation. The increase in the solubility of ketoprofen was achieved by the inclusion of citric acid (the amount of citric acid incorporated was found to be osmotically inactive) in the formulation because, unlike a conventional dose, the formulations without citric acid were not able to achieve therapeutic concentrations within the first hour, probably owing to the lower solubility of ketoprofen in the acidic medium. The incorporation of citric acid in the formulation provided an increased microclimate pH of stagnant diffusion layer around the drug particle, which was around the pKa of ketoprofen (∼4)^16^. This stagnant diffusion layer was at a higher pH than the bulk of the dissolution medium (SGF, pH 1.2). Because higher pH favors the dissolution of weakly acidic drugs, the solubility of ketoprofen increased in the stagnant diffusion layer at a higher pH, thereby resulting in a higher release from the formulation as compared with other formulations without citric acid.

**Fig. 1:**
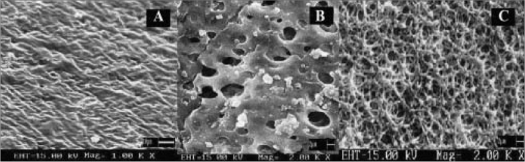
Scanning electron microscope photographs of the *in situ* formed AMC Scanning electron microphotographs of coating membrane obtained A) before dissolution, showing outer dense nonporous region and containing 8% w/v glycerol at 1000 X, B) before dissolution showing large inner porous region and containing 12% w/v glycerol at 2000 X, C) after complete dissolution showing net like structure and containing 8% w/v glycerol at 2000 X.

For SEM studies, 15% w/v EC membranes with varying proportions, as mentioned above of pore forming agent (glycerol) were obtained before and after complete dissolution. SEM revealed, membrane obtained before dissolution (8% w/v glycerol) had an outer dense non porous region (fig. 1 A) and an inner lighter porous region. After complete dissolution, the exhausted membrane showed large number of pores similar to a net like structure (fig. 1 C) and formulation prepared with this membrane did not show swelling or rupturing. Membrane containing 12% w/v of glycerol showed similar nonporous but larger inner porous regions (fig. 1 B) with swelling or elongation and slight rupture at the end of 7 h of dissolution study. Membrane containing higher proportion of glycerol (20% w/v) showed larger pores and formulation prepared with this membrane caused bursting within an hour of dissolution study. SEM studies suggested concentration of plasticizer to be an important parameter in deciding membrane strength as, high concentrations of glycerol made asymmetric membrane correspondingly weak and then caused rupturing. Membranes with 12%,16% and 20% w/v glycerol showed flimsy nature (which could account for their rupturing during dissolution) during preparation process which, involved pouring of coating solution inside hard gelatin capsule but were hardened during drying process. These results could probably be due to presence of plasticizer in hard gelatin capsule itself which might infact add up to the concentration of plasticizer present in asymmetric membrane inside hard gelatin capsule. Based on SEM study, 8% w/v glycerol as a plasticizer was selected for further studies.

**Fig. 2:**
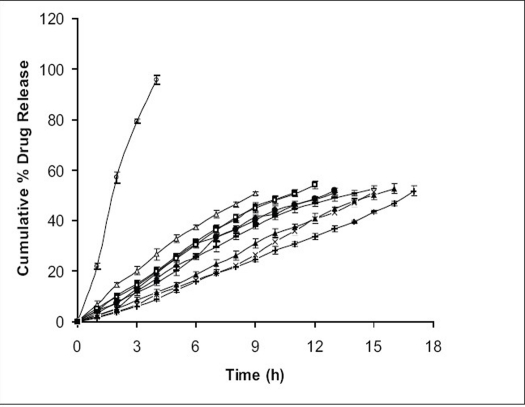
Comparative *in vitro* dissolution profiles Comparative *in vitro* dissolution profiles (n=3 along with standard deviations) for all formulations along with the marketed formulation. F1 (–◆–); F2 (–■–); F3 (–Δ–); F4 (–×–); F5 (–●–); F6 (–□–); F7 (– ‌ –); F8 (–▲–) F9 (–––); Marketed Formulation (–◊–)

**Fig. 3:**
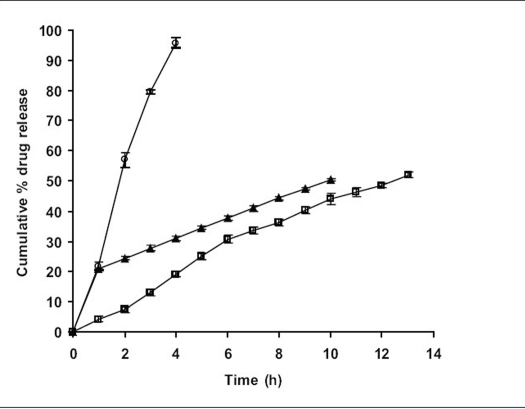
Comparative dissolution profile of F5, F10 and F11 Comparative *in vitro* dissolution profiles (n=3 along with standard deviations) for F5 (–●–), marketed formulation, F10 (–◊–) and formulation with solubility enhancer for the drug, F11 (–▲–).

*In vitro* studies were carried out for all the prepared formulations along with the marketed formulation (fig. 2) and the solubility enhancer for the drug (fig. 3). Order of influence for t_50%_ of F1, F2 and F3 formulations with concentration of EC at lower levels (10% w/v) were F1 (13.16 h) > F2 (11.14 h) > F3 (9.26 h). Descending order of influence meant, incorporation of NaCl in F2 and F3 results in development of significant osmotic pressure inside the capsular system, creating an osmotic pressure gradient between inner portion of the capsular system and dissolution medium, which, increased the release rate of ketoprofen far more than F1 formulation, even though ketoprofen had poor solubility in SGF suggesting, release of ketoprofen from *in situ* formed AMC to be independent of pH. Order of influence for t_50%_ of F4, F5 and F6 formulations with concentration of EC at medium level (15% w/v) were F4 (15.02 h) > F5 (12.50 h) > F6 (11.32 h). Here once again, influence of NaCl in increasing release rate from *in-situ* formed phase transited AMC was demonstrated even though, osmotic effect due to osmogen was constrained. Decreased ketoprofen release from these formulations as compared to F1, F2 and F3 formulations might probably be due to increased diffusional path for drug to traverse before being released into the dissolution medium. Order of influence for t_50%_ of F7, F8 and F9 formulations with concentration of EC at higher level (20% w/v) were F7 (16.42 h) > F8 (15.190 h) > F9 (14.23 h) suggesting, influence of NaCl again, in drug release though here the influence of EC concentration at higher level was substantial in constraining drug release probably due to increased drug holding capacity for the polymer.

Statistical analysis was performed on all formulations undergoing *in vitro* dissolution study using Dunnett's Multiple Comparison Test. Dissimilarity factor (f_1_) given in SUPAC guidelines for modified dosage forms was used to further justify the selection of best formulation which showed least significance during multiple t-test at P>0.05. Dunnett's Multiple Comparison Test compared all the formulation with the marketed formulation of ketoprofen (F10). Values calculated for all formulations (q<2.619, F=0.2789 and *P*=0.9459) suggested, the test was run at 0.05 significance level or 95% confidence level and that difference between all formulations as compared to the marketed formulation (F10) were statistically insignificant, since if value of q was greater than 2.659, then comparison test would have run at a significance value less than 0.05 or below 95% confidence level and would have been considered to be statistically significant. Dissimilarity factor (f_1_) was calculated between all formulations and F10. Dissimilarity factor (f_1_) between F5 and F10 was found to be 4.2 (q=0.04356), which was lowest with zero order kinetics amongst all formulations compared to F10, suggesting, the two formulations (F5 and F10) to have completely different dissolution profiles.

**Fig. 4:**
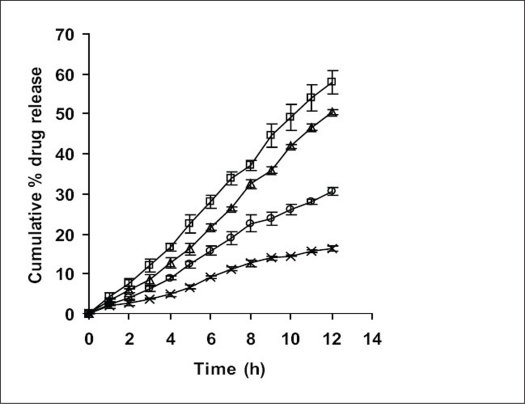
Comparative release profiles of F5 in mediums of different osmotic pressure Comparison of *in vitro* ketoprofen release profiles (n=3 along with standard deviations) from F5 in dissolution medium of different osmotic pressures. F-5a (–□–) (3.673 mm Hg), F-5b (–Δ–) (7.348 mm Hg), F-5c (–◊–) (11.012 mm Hg) and F-5d (–×–) (14.695 mm Hg).

**Fig. 5:**
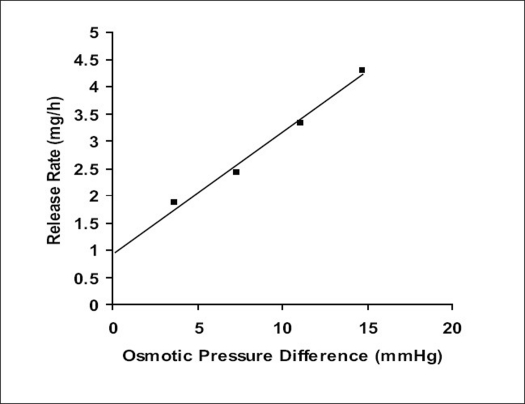
Demonstration of osmotic pressure Ketoprofen release rate from F5 showing effect of osmotic pressure difference across the membrane.

Since the study was based on osmotic delivery therefore, to study effect of varying osmotic pressure, release studies of optimized formulation F5 were conducted in media of different osmotic pressures (fig. 4). Cumulative % drug release after 12 h from F-5a was found to be 57.92±2.83, from F-5b i	t was 50.36±0.65, from F-5c it was 30.59±0.93 and from F-5d it was 16.38±0.71. Results of release studies suggested, drug release to be highly dependant on osmotic pressure of release media. Ketoprofen release from F5 decreased as osmotic pressures of release medium increased. R^2^ of 0.9873 from linear line obtained when release rate was plotted against osmotic pressure difference (osmotic pressure inside the formulation was found to be 18.369 mm Hg) shown in fig. 5 suggested, osmotic pumping as primary mechanism governing drug release from the developed formulations.

The analytical performance parameters namely specificity, linearity, range, precision, accuracy, limit of detection and limit of quantification were validated according to International Conference on Harmonization ICH Q2B guidelines. Specificity was assessed by comparing the chromatograms obtained from the drug to their respective internal standards and with those obtained from the blank which verified the absence of any interference. The linearity of the method used for the drug was evaluated on a standard curve of the peak area versus the concentration of the analyte. A five point calibration curve was constructed with working standards and was found linear (R^2^ = 0.9999) in a range 0.3-10 μg/ml (R_t_ were found to be 3.36±0.09 min and 3.12±0.05 min for ketoprofen and the internal standard respectively). The LOD and LOQ were found to be 0.10 μg/ml and 0.30 μg/ml respectively. The results of determination of accuracy using quality control concentrations (QC) were 99.96±0.05%. Precision assay showed that the averages of the relative standard deviations within 1 day (intraday) were 0.87-3.12% and among every other day (interday) were 0.98-2.88%. The results showed that the method was accurate. Recovery test was performed again on QC samples and the results, 99.77±1.23 μg/ml for 1 μg/ml, 99.25±0.89 for 5 μg/ml and 100.33±0.15 for 10 μg/ml, validated the method.

**Fig. 6:**
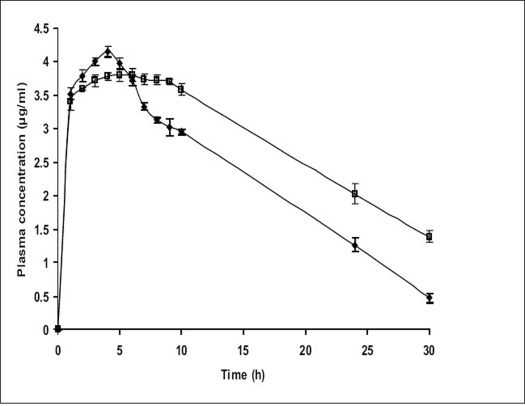
*In vivo* release profile of test and reference formulations Comparative plasma concentration time profile (n =3 along with standard deviation) of test formulation (–□–) and reference formulation (–◆–).

The relevant pharmacokinetic parameters are listed in Table 2. From fig. 6, it is apparent that the specially designed dosage form effectively sustained and controlled the release of ketoprofen and also maintained elevated plasma concentrations up to the 10^th^ h. Although the statistical analysis of the C_max_ and T_max_ values for the conventional tablets and the test AMCs were statistically insignificant (*P*>0.01) at 99% confidence level, there was statistically significant difference (*P*<0.01) between the elimination half life and AUC for the two formulations of the drug. This suggested the capacity of the test formulation to sustain the release of drug. The reduction in AUC for the conventional tablet could be interpreted as that the formulation with a rapid rate of drug release tends to attain lower systemic bioavailability. This observation could be attributed to the fact that as the drug releases at a rate that exceeds the rate of absorption; it leads not only to side effects but also to first pass metabolism. Test formulations with increased AUC could be attributed to sustained drug release at a lower rate thus not only enhancing the bioavailability which meant enhanced absorption (% relative bioavailability showed an improvement of 39.24%), but also minimizing the first pass metabolism.

**TABLE 2:** VARIOUS PHARMACOKINETIC PARAMETERS CALCULATED USING QUICK-CAL SOFTWARE

Parameters	Reference tablets for ketoprofen	Test formulation for ketoprofen
C_max_ (h)	4.15±0.09	3.80±0.11
T_max_ (h)	4.00±0.10	6.00±0.09
t1/2 (el) (h)	9.03±0.67	16.49±0.28
K_a_ (h)	0.67±0.23	0.45±0.17
K_el_ (h)	0.08±0.01	0.04±0.07
V_d_ (l/kg)	1.30±0.43	6.08±0.34
Cl (l/kg h)	0.10±0.12	0.26±0.13
AUC_0-t*_ (h.μg/ml)	68.76±0.21	88.50±0.33
AUC_t*-∞_ (h.μ/ml)	6.12±0.31	29.04±0.29
Relative Bioavailability (%)	----	39.24±0.04

Cmax- Peak concentration, T_max_- Time to reach peak concentration,_t1/2 (el)_- Elimination half life, K_a_- First order absortion rate constant, K_el_- First order elimination rate constant, V_d_- Volume of distribution. Cl- Clearance, AUC-Area under the curve

**Fig. 7:**
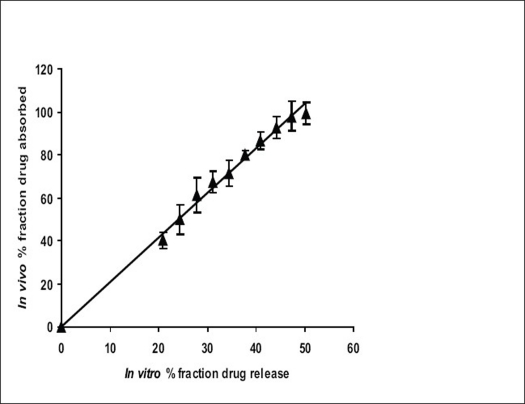
Level A *in vitro in vivo* correlation *In vitro in vivo* correlation of in situ formed asymmetric membrane capsule delivering ketoprofen.

The results of IVIVC (fig. 7) demonstrated that a good level A correlation (R^2^ = 0.9927) could be achieved with the fabricated *in situ* formed phase transited AMCs between the fraction of drug release from the dosage units and the fraction of drug absorbed. Therefore, the *in vitro* release profile of ketoprofen from the fabricated asymmetric membrane capsules could be used to accurately predict their *in vivo* performance.

In conclusion, *in situ* formed phase transited AMCs of ketoprofen were successfully prepared and tested both *in vitro* and *in vivo*. The phase transited drug delivery system not only showed level A correlation but also controlled release with osmotic pumping as the principle mechanism of release. Level A correlation meant that by using *in vitro* release profile of ketoprofen from *in situ* formed phase transited AMC, one can also predict their *in vivo* performance.
